# The Effects of Yoga on Key Adipocytokines in Obesity: A Narrative Review of Leptin and Adiponectin

**DOI:** 10.7759/cureus.76792

**Published:** 2025-01-02

**Authors:** Rinne Shimizu, Hajime Suzuki, Marie Amitani, Haruka Amitani

**Affiliations:** 1 Department of Psychosomatic Internal Medicine, Kagoshima University Graduate School of Medical and Dental Sciences, Kagoshima, JPN; 2 Kampo Medical Center, Kagoshima University Hospital, Kagoshima, JPN; 3 Department of General Medicine, Ryokusenkai Yonemori Hospital, Kagoshima, JPN; 4 Department of Oral and Maxillofacial Surgery, Kagoshima University Graduate School of Medical and Dental Sciences, Kagoshima, JPN

**Keywords:** adipocytokines, adiponectin, chronic inflammation, hormonal regulation, leptin, lifestyle intervention, obesity management, yoga

## Abstract

Obesity is a global health concern that increases the risk of numerous complications, including type 2 diabetes, hypertension, and cardiovascular diseases. Conventional obesity treatments, such as lifestyle modifications, pharmacotherapy, and surgical interventions, are often insufficient, highlighting the need for more efficient and effective approaches. Yoga, an ancient mind-body practice incorporating physical postures (asanas), breathing exercises (pranayama), and meditation, has emerged as a potential therapeutic intervention for obesity management. This review examines the functions of leptin and adiponectin, two key adipocytokines central to obesity, and evaluates the impact of yoga on these hormones. A literature search was conducted using PubMed, Scopus, and Google Scholar with the keywords "yoga" and "adipocytokine" as of May 5, 2024, resulting in the selection of 12 relevant studies. The majority of studies reviewed demonstrated that yoga significantly decreases leptin levels and increases adiponectin levels. Intensive yoga sessions and combined dietary interventions were found to contribute notably to improvements in these hormonal levels. These findings suggest that yoga may improve the balance between leptin and adiponectin, offering beneficial effects on anti-obesity and chronic inflammation reduction. Yoga, as an economical and non-invasive treatment option, presents a promising approach to managing obesity. Further research is expected to elucidate the underlying mechanisms and explore potential clinical applications.

## Introduction and background

Obesity is a major health problem worldwide. People with obesity often develop serious health disorders like type 2 diabetes, hypertension, and dyslipidemia [1-3]. Obesity increases the risk of malignancy, infections, ischemic heart disease, and pulmonary embolism, contributing to significant morbidity and mortality worldwide [4].

The pathophysiology of obesity primarily manifests as excessive visceral fat accumulation due to chronic energy imbalance between intake and expenditure. This visceral adipose tissue functions not merely as an energy storage depot but as an active endocrine organ, secreting numerous bioactive substances called adipocytokines that regulate metabolic and immune responses. Among these adipocytokines, leptin and adiponectin play crucial roles in metabolic regulation. Leptin helps control appetite and energy expenditure through its actions on the hypothalamus. However, in obesity, despite elevated leptin levels, the body often becomes resistant to its effects, leading to disrupted appetite regulation and energy metabolism. In contrast, adiponectin, which has anti-inflammatory properties, is typically decreased in obesity. This reduction in adiponectin levels compromises its protective effects against inflammation and metabolic dysfunction. These adipocytokine disturbances collectively contribute to the development of chronic low-grade inflammation, which in turn causes two major pathophysiological changes: insulin resistance and endothelial dysfunction. These conditions are fundamental mechanisms underlying obesity-related disorders such as type 2 diabetes and cardiovascular disease [5].

Current treatments for obesity mainly focus on lifestyle changes, such as dietary restriction and exercising more. However, these lifestyle changes alone often show limited effectiveness, especially in people with chronic obesity. While additional treatment options exist, such as pharmacological treatments including glucagon-like peptide-1 (GLP-1) receptor agonists and surgical interventions, their use is often limited to specific patients due to high costs, limited accessibility, and potential side effects [6]. Therefore, there is a critical need for cost-effective, easily accessible, safe, and effective therapeutic options for obesity management.

Yoga is an ancient mind-body practice. It combines physical movement, breathing exercises, and meditation, making it a whole-body approach to health [7]. Traditionally, yoga aims to harmonize the body, mind, and spirit, promoting overall well-being. As a treatment option, yoga has several advantages: it is generally safe, does not require expensive equipment, and can be adapted for different fitness levels [8].

This review examines how yoga might affect leptin and adiponectin levels in people with obesity. Previous studies examining yoga's effects on these adipocytokines have several limitations: small sample sizes, inconsistent yoga protocols, and short intervention periods. Despite these limitations, several studies have suggested potential benefits. This review synthesizes available research about the effects of yoga on leptin and adiponectin, examining its potential as a complementary approach to existing obesity treatments, particularly for those who have limited access to conventional treatment options.

## Review

Chronic inflammation in obesity and leptin

Leptin is a hormone derived from the obesity gene discovered through research on the pathogenic gene in genetically obese mice. It is primarily secreted by adipose tissue and exerts potent appetite suppression by binding to and activating the long form of its membrane receptor (LEPR-B), which is prominently expressed in the hypothalamus and other brain regions, influencing food intake and energy expenditure. The leptin receptor Ob-Rb shares high homology with gp130, a signaling molecule for inflammatory cytokines, and is known to be expressed in peripheral tissues such as macrophages [9]. Thus, leptin may also act as an inflammatory cytokine in peripheral tissues. Furthermore, studies have shown that leptin-deficient mice and leptin receptor mutant mice exhibit suppressed thrombus formation and neointimal formation after arterial injury, suggesting that leptin may be involved in post-vascular injury remodeling [10]. In other words, leptin is essential for controlling inflammatory responses and maintaining cardiovascular homeostasis and is thought to be involved in the increase of inflammation not only in obesity but also in cardiovascular disease and type 2 diabetes [11].

Leptin resistance

Leptin levels correlate strongly with body fat, as increased adipocytes trigger a rise in leptin to regulate energy balance [12]. However, many obese individuals develop leptin resistance, where hypothalamic neurons become less sensitive or unresponsive to high leptin levels, leading to increased calorie intake and difficulty maintaining weight loss [13,14].

The primary cause of leptin resistance is impaired leptin transport across the blood-brain barrier (BBB). Studies in high-fat diet-induced obese (DIO) rats have shown reduced BBB permeability to leptin despite elevated plasma leptin concentrations [15].

The second cause of leptin resistance is impaired leptin signaling in neurons. Obese mice show increased expression levels of suppressor of cytokine signaling 3 (SOCS3), a negative feedback regulator of leptin signaling, which has been shown to induce leptin resistance [16,17]. Analysis using protein tyrosine phosphatase 1B (PTP1B) knockout mice has demonstrated that PTP1B is also a negative regulator of leptin signaling and contributes to the development of leptin resistance [18,19]. Furthermore, PTPRJ (protein tyrosine phosphatase receptor type J) is co-expressed with leptin receptors in hypothalamic neurons and negatively regulates leptin signaling. PTPRJ-deficient mice exhibit enhanced leptin signaling and reduced food intake, resulting in less weight gain compared to wild-type mice. Diet-induced obesity and leptin administration increase PTPRJ expression in the hypothalamus, and PTPRJ overexpression induces leptin resistance. Thus, PTPRJ induction is implicated in the development of leptin resistance, and PTPRJ inhibition may be a potential strategy for improving obesity [20].

Another mechanism involves hypothalamic inflammation, endoplasmic reticulum stress, and autophagy defects [21,22]. Obese mice show increased endoplasmic reticulum stress [23]. Neuronal treatment with stress-inducing agents like tunicamycin, thapsigargin, and brefeldin A inhibits leptin-induced STAT3 activation, which can be reversed by 4-phenylbutyric acid, a chemical chaperone. This suggests that abnormal protein accumulation from endoplasmic reticulum stress contributes to leptin resistance [24,25]. Short-term high-fat feeding promotes microglial infiltration and hypothalamic inflammation. Chronic high-fat feeding exacerbates endoplasmic reticulum and oxidative stress, enhancing IKKβ/NF-κB signaling and creating a cycle that worsens leptin resistance through SOCS-3 induction. This signaling is further amplified by endoplasmic reticulum stress. Intracerebroventricular administration of tauroursodeoxycholic acid (TUDCA), a molecular chaperone, suppresses NF-κB signaling under chronic high-fat conditions [26]. Both oxidative and endoplasmic reticulum stress in the hypothalamus impairs autophagy, increasing IKKβ/NF-κB signaling [27].

Medical applications of leptin

Leptin has been anticipated as a potential obesity treatment, but its clinical application has not become widespread. Current pharmacological and behavioral therapies for obesity induce initial weight loss, but this effect is transient due to leptin resistance [28]. Leptin resistance also reduces the effectiveness of exogenous leptin therapy in obese individuals [29].

Leptin gene therapy is emerging as a new treatment option. It has improved type 1 and type 2 diabetes and diet-induced obesity in animal models, including ob/ob mice, DIO mice, and insulin-deficient diabetic mice. A single central administration of a recombinant adeno-associated virus vector encoding the leptin gene significantly reduced adiposity and improved metabolic syndrome (MetS) symptoms in various models over an extended period. In contrast, obese patients with hyperleptinemia, with or without type 2 diabetes, did not respond to exogenous r-metHuLeptin [22]. Leptin gene therapy is expected to become an effective treatment option for obesity and diabetes. Nevertheless, several challenges remain in the clinical application of leptin gene therapy. For instance, the development of leptin resistance and impaired transport of leptin across the BBB may limit its therapeutic efficacy. Additionally, considerations regarding the safety of viral vectors, the maintenance of long-term efficacy, and the potential risk of immune responses associated with gene therapy must be addressed. Further research is required to overcome these challenges.

A 2024 report by Francesca Mainieri et al. suggested that leptin gene therapy could be effective in treating genetic obesity caused by loss-of-function mutations in genes involved in the leptin-melanocortin pathway [30]. These loss-of-function mutations in genes involved in the leptin-melanocortin pathway are considered one of the causes of leptin resistance, and with future advances in genetic analysis, innovative pharmacological treatments for various genetic obesities are anticipated.

Additionally, the gut-brain axis has been identified as a new mechanism for improving leptin resistance in obesity. The gastrointestinal-brain axis refers to the mechanism through which various signaling molecules secreted by the gastrointestinal tract act via the vagus nerve and circulatory pathways to influence the central nervous system, particularly regions such as the hypothalamus and the dorsal motor nucleus of the vagus nerve, to regulate appetite and energy balance. This axis mediates the effects of gastrointestinal-derived hormones, such as ghrelin, on satiety and feeding behavior [31]. Approaches targeting the gastrointestinal-brain axis, by enhancing leptin sensitivity through signals secreted from the digestive tract, offer promise as adjunctive therapeutic strategies. Various anorexigenic signals secreted from the gastrointestinal tract that act on specific brain regions such as the hypothalamus, including uroguanylin, GLP-1, amylin, and cholecystokinin, may alleviate resistance to leptin's effects by promoting the secretion of appetite-suppressing signals [32]. A 2014 report by Folgueira et al. demonstrated that various anorexigenic signals secreted from the gastrointestinal tract, together with leptin, induced significant weight loss even in DIO mice, a model of leptin resistance [33]. Further elucidation of the gut-brain axis is expected to become a new option for obesity treatment through leptin resistance. This approach suggests the potential of targeting the gastrointestinal-brain axis as an adjunctive therapeutic modality for addressing leptin resistance.

Chronic inflammation and adiponectin in obesity

Adiponectin, despite being specifically secreted by adipocytes, decreases in obesity and visceral fat accumulation [34]. It has been shown to have anti-diabetic, anti-atherosclerotic, and anti-inflammatory properties. In obese and type 2 diabetic mouse models, decreased plasma adiponectin levels lead to insulin resistance and dyslipidemia, but appropriate administration of adiponectin improved these pathological conditions [35]. Furthermore, in patients with type 2 diabetes and coronary artery disease matched for body mass index (BMI), blood adiponectin levels were found to be reduced [36].

Medical applications of adiponectin

In 2023, Naomi Asahara et al. reported obtaining an antibody that activates adiponectin receptors similarly to adiponectin itself (Adiponectin Receptor Activating Monoclonal Antibody: AdipoRaMab). AdipoRaMab binds to adiponectin receptors (Adiponectin Receptor 1: AdipoR1 and Adiponectin Receptor 2: AdipoR2), promoting the phosphorylation of adenosine monophosphate-activated protein kinase (AMPK), which activates pathways involved in glucose uptake and oxidative metabolism, thereby enhancing insulin sensitivity in muscle and liver tissues. Additionally, AdipoRaMab suppresses the expression of inflammation-related factors, such as interleukin-6 (IL-6) and monocyte chemoattractant protein-1 (MCP-1), in the liver while increasing the expression of genes related to energy expenditure. These effects contribute to the amelioration of chronic inflammatory conditions, such as non-alcoholic fatty liver disease (NAFLD), including fatty hepatitis [37]. This discovery suggests the potential for treating chronic diseases caused by reduced adiponectin function, beyond diabetes treatment, and future research developments are anticipated.

Regulation of adipocytokines through yoga

**Figure 1:**
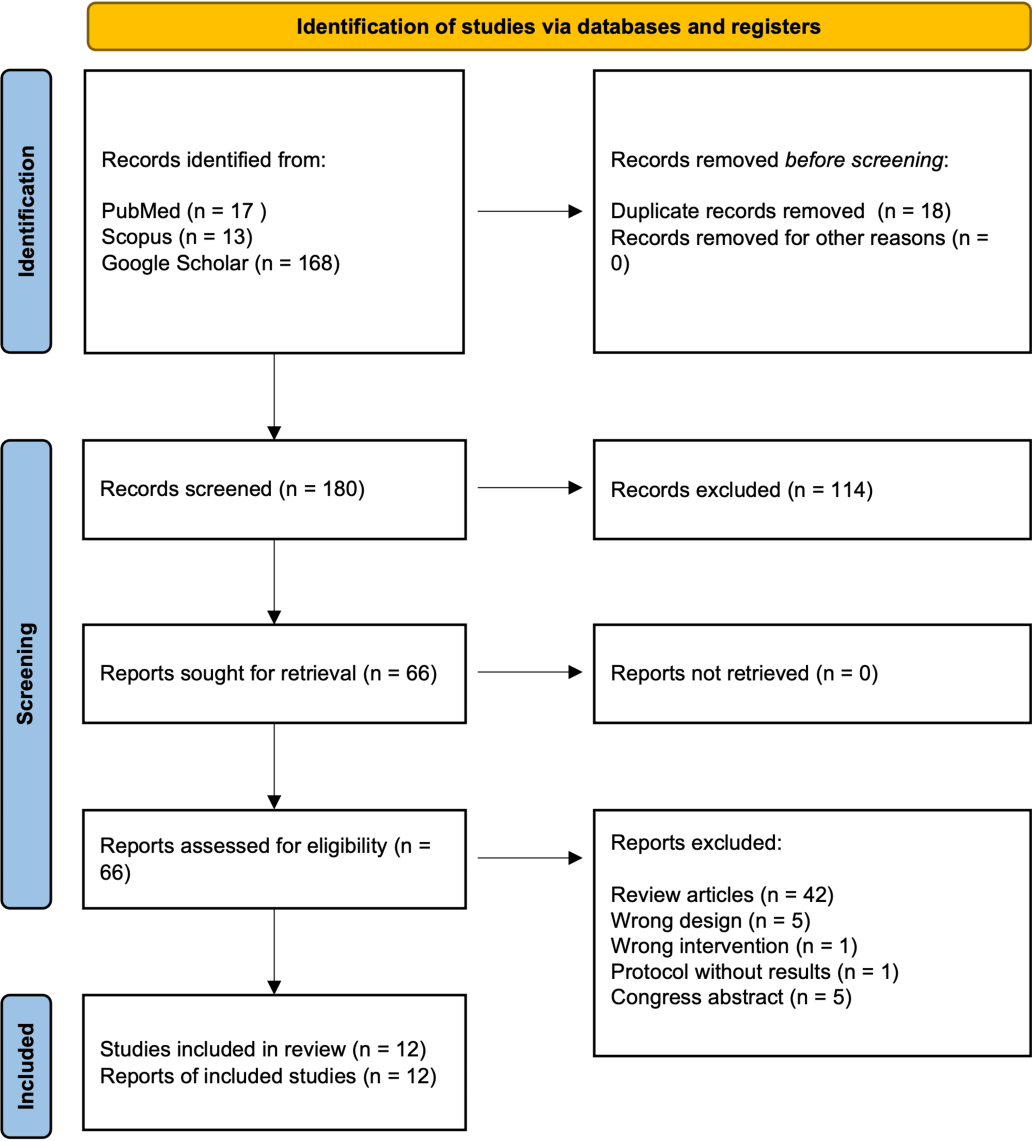
PRISMA 2020 Flow Diagram The flowchart illustrates the selection process of studies included in this narrative review. Initially, 198 records were identified through database searches, and after removing duplicates and screening titles/abstracts, 66 records were assessed for full-text eligibility. Following further screening based on the inclusion/exclusion criteria, a total of 12 studies were included in the qualitative synthesis.

Yoga emphasizes lifestyle improvement and increased physical activity. It has been shown to be effective in weight reduction and improving lipid profiles in patients with coronary artery disease, diabetes, and hypertension [6,38-40]. In this narrative review, a literature search was conducted on May 5, 2024, using the keywords "yoga" and "adipocytokine" in major medical literature databases, including PubMed, Scopus, and Google Scholar. A PRISMA 2020 flow diagram was used to illustrate the study selection process (Figure 1). 

Initially, a total of 198 records were identified through database searches in PubMed (n=17), Scopus (n=13), and Google Scholar (n=168). After removing duplicates, 180 records remained. These records were screened based on their titles and abstracts, leading to the exclusion of 114 records for the following reasons: studies without yoga interventions (n=72), irrelevant studies (n=35), and studies focused on gynecological conditions (e.g., polycystic ovary syndrome, breast cancer, and pregnancy) (n=7). This screening process resulted in 66 records being assessed for full-text review. Of these 66 full-text articles, 54 were excluded for the following reasons: review articles (n=42), editorials/posters/reports (n=5), observational studies (n=4), a cross-sectional study (n=1), a protocol paper (n=1), and combined interventions with other exercises (n=1). Ultimately, 12 studies [41-52] were included in the qualitative synthesis for this review (Table 1). 

**Table 1:** Summary of Studies Examining the Effects of Yoga on Adipokines and Health Parameters This table summarizes findings from studies evaluating the effects of yoga interventions on adipokines, including leptin, adiponectin, IL-6, visfatin, and chemerin, as well as associated metabolic health parameters across various populations. The included studies encompass a range of designs, such as randomized controlled trials, quasi-experimental studies, and pre-post-intervention studies. Participant groups included individuals with obesity, MetS, type 2 diabetes, and healthy individuals. Yoga protocols varied in duration, frequency, and integration with dietary modifications. Key findings highlight reductions in leptin levels and increases in adiponectin levels, often accompanied by improvements in metabolic markers such as BMI, blood glucose, and lipid profiles (e.g., cholesterol). In several studies, dietary interventions were employed, further potentiating the effects of yoga on adipokine regulation and overall metabolic health. 8-OHdG, 8-hydroxy-2'-deoxyguanosine; BMI, body mass index; HDL, high-density lipoprotein; HOMA-IR, homeostasis model assessment of insulin resistance; IL-6: interleukin-6; LDL, low-density lipoprotein; MetS, metabolic syndrome; NA, not available; PAI-1: plasminogen activator inhibitor-1; SOD, superoxide dismutase; TBARS: thiobarbituric acid reactive substances

Author/year	Study design	Participants/sample size	Yoga protocol	Diet change advised	Adipokines assessed	Key findings (yoga intervention group）
Ritesh N, et al./2015 [39]	Pilot study	Overweight or obese adults (BMI 23-35 kg/m²)/n=34	10 days yoga program	Advised healthy eating patterns	IL-6, vaspin	Decrease in IL-6, neopterin, vaspin, body weight, BMI, fasting blood glucose, fasting insulin, HOMA-IR, total cholesterol, triglyceride, BMI, waist and hip circumferences.
Daneshyar, et al./2020 [40]	Quasi-experimental study	Postmenopausal women/n=33	30-40 minutes, 3 sessions per week, 8 weeks yoga program,	NA	Visfatin	No changes in visfatin, liver enzymes, or body composition.
Supriya R, et al./2018 [41]	Randomized controlled trial	MetS and high-normal blood pressure/n=97	1 hour, 3 times per week for 1 year	NA	Leptin, adiponectin, chemerin, visfatin	Decreases in leptin and chemerin. Increase in adiponectin. No change in visfatin, PAI-1.
Telles S, et al./2010 [42]	Single group pre-post intervention study	Obese persons/n=47	5 hours, every day for 6 days	Low fat, high fiber, vegetarian diet	Leptin	Decrease in leptin, total cholesterol, HDL cholesterol, BMI, waist and hip circumferences, fat-free mass.
Yadav R, et al./2019 [43]	Randomized controlled trial	WetS/n=260	12 weeks yoga program	Advised healthy eating patterns	Leptin, adiponectin, leptin/adiponectin ratio, IL-6	Decrease in leptin, leptin/adiponectin ratio, IL-6, TBARS, 8-OHdG. Increase in adiponectin, SOD.
Telles S, et al./2010 [44]	Single-group pre-post interventional study	Type 2 diabetes/n=31	60-90 minutes, twice a day	Plant-based diet (1800 kcal/day)	Leptin, adiponectin	Decrease in leptin, fasting blood glucose and cholesterol.
Arciero PJ, et al./2014 [45]	Randomized controlled trial	Overweight or obese adults (BMI 25-35 kg/m²)/n=79	PRISE group (protein + resistance training + intervals + stretching/yoga/pilates + endurance): Stretching/yoga/pilates session once a week	Whey protein protocol	Leptin, adiponectin	Decrease in leptin, HOMA-IR, visceral adipose tissue, body weight and fat mass. Increase in adiponectin.
Farideh Y, et al./2020 [46]	Randomized controlled trial	Obese or overweight women/n=44	8 weeks yoga program	Energy-restricted diet of 300 kcal/day	Leptin, adiponectin	Decrease in leptin. Increase in adiponectin.
Lee JA, et al./2012 [47]	Randomized controlled trial	Postmenopausal women, body fat ≥36%/n=16	NA	NA	Adiponectin	Decrease in total cholesterol, triglyceride, LDL, blood pressure, insulin, glucose, and HOMA-IR, body weight, percentage of body fat, lean body mass, BMI, waist circumference, and visceral fat area. Increase in adiponectin, HDL.
Sarvottam K, et al./2013 [48]	Nonrandomized pre-post intervention study	Overweight and obese men (BMI 26.26±2.42 kg/m^2^)/n=51	NA	NA	Adiponectin, IL-6	Decreases in IL-6, weight, BMI, and systolic blood pressure. Increase in adiponectin.
Papp ME, et al./2016 [49]	Randomized controlled trial	Healthy students/n=44	60 minutes, once a week for 6 weeks	Low fat, high fiber, vegetarian diet	Leptin, adiponectin	No change in leptin. Increase in adiponectin and ApoA1.
Telles S, et al./2014 [50]	Randomized comparative trial	BMI ≥25 kg/m^2^/n=68	45 minutes, twice daily	Plant-based diet (1650 kcal/day)	Leptin, adiponectin	Decrease in cholesterol, LDL, lean mass, body water. Increase in leptin.

The most frequently studied adipocytokine was adiponectin, assessed in nine studies, followed by leptin, examined in eight studies. Other adipocytokines, including IL-6, vaspin, visfatin, chemerin, and PAI-1, were also evaluated. Additionally, some studies measured neopterin, a biomarker indicative of immune system activation related to glucose and lipid metabolism, as well as oxidative stress markers such as thiobarbituric acid reactive substances (TBARS) and 8-hydroxy-2'-deoxyguanosine (8-OHdG).

Nine of the studies involved subjects with obesity-related conditions such as overweight, MetS, or type 2 diabetes. Two studies focused on healthy individuals. Of the eight studies examining leptin, six reported a decrease in leptin levels. Among the nine studies investigating adiponectin, seven observed an increase in adiponectin levels. Nine studies showed favorable regulation of either leptin or adiponectin, with six of these studies incorporating dietary guidance. Dietary interventions included advised plant-based diets (1650-1800 kcal/day), vegetarian diets, and whey protein supplementation.

Reduction of leptin levels through yoga

In a 2018 study by Supriya R, et al., subjects with MetS and high-normal blood pressure, with a mean age of 57.6±9.1 years, practiced yoga for one year. As a result, leptin levels decreased by approximately 26.5%, from an average of 22.2 ng/mL to 16.3 ng/mL [43].

Telles et al. reported in 2010 that obese individuals aged 17-68 years with a BMI ≥30 kg/m² participated in a six-day yoga and dietary modification program. Leptin levels decreased by about 44.2%, from an average of 53.71 ng/mL to 29.95 ng/mL [44].

Yadav et al. examined Indian adults aged 20-45 years with MetS. After a 12-week yoga and lifestyle intervention program, leptin levels decreased by approximately 16.4%, from 57.49±18.89 ng/mL to 48.08±16.68 ng/mL. The yoga program included asanas (postures), pranayama (breathing techniques), meditation, and shavasana (relaxation technique), along with individualized lifestyle counseling. The recommended diet included 50-60% carbohydrates, <30% total fat, <10% saturated fat, 10-15% protein, 25-40g/day fiber, and <5g/day salt [45].

In another 2010 study by Telles et al., patients with uncomplicated type 2 diabetes (mean age 47.8±6.2 years) participated in a six-day yoga and dietary modification program for five hours daily. Leptin levels decreased significantly by about 64.1%, from 20.72±22.55 ng/mL to 7.44±7.38 ng/mL [46].

Arciero et al. studied overweight or obese individuals aged 35-65 years with BMI ≥25 kg/m². After 16 weeks of yoga practice and whey protein intake, leptin levels decreased by approximately 35.2%, from 37.5±15.7 ng/mL to 24.3±10.2 ng/mL [47].

Yazdanparast et al. reported in 2020 that overweight and obese women aged 30-50 years with BMI ≥25 kg/m² participated in an eight-week yoga and dietary modification program. Leptin levels decreased by about 16.4%, from 57.49 ng/mL to 48.08 ng/mL [48].

These studies suggest that yoga significantly reduces leptin levels. Notably, Supriya et al. demonstrated sustained leptin reduction over a longer period [43], supporting the efficacy of long-term yoga practice in managing MetS and obesity. Conversely, a short-term intensive intervention by Telles et al. (five hours daily for six days) in patients with uncomplicated type 2 diabetes showed a remarkable 64.1% decrease [46], indicating the potential for powerful effects with intensive, short-term yoga practice. The studies also showed enhanced effects when yoga was combined with dietary modifications or whey protein intake [44-48].

Increase in adiponectin levels through yoga

Supriya et al. found that in individuals with MetS and high-normal blood pressure (mean age 57.6±9.1 years), one year of yoga practice increased adiponectin levels by about 20.1%, from 6.48±1.80 µg/mL to 7.77±2.12 µg/mL [43]. Yadav et al. examined Indian adults with MetS (aged 20-45 years) and found a 7% increase in adiponectin levels, from 5.18±1.39 µg/mL to 5.54±2.41 µg/mL, after a 12-week yoga and lifestyle intervention program [45]. Arciero et al. investigated overweight or obese individuals (aged 35-65 years, BMI ≥25 kg/m²) and found a 22.9% increase in adiponectin levels, from 18.8±1.90 µg/mL to 23.1±3.10 µg/mL, after 16 weeks of yoga practice and whey protein intake [47]. Farideh et al. evaluated overweight and obese women (aged 30-50 years, BMI ≥25 kg/m²) and observed a 12.0% increase in adiponectin levels, from 8.35±2.85 μg/mL to 9.35±3.30 μg/mL, after an eight-week yoga and dietary modification program [48]. Lee et al. investigated postmenopausal obese women (mean age 54.5±2.75 years) with body fat percentage ≥36% and found an increase in adiponectin levels by approximately 16.5%, from 6.60±2.08 µg/mL to 7.68±2.420 µg/mL [49]. Sarvottam et al. studied overweight or obese men (mean age: 39±12.2 years, BMI: 23-35 kg/m²) in a 10-day program of two-hour daily yoga sessions with dietary modifications [50]. Adiponectin levels increased by about 26.5%, from 4.95±1.39 µg/mL to 6.26±2.41 µg/mL. The yoga program included asanas, pranayama, group discussions, lectures, and individual counseling. A predominantly plant-based diet was recommended. Papp et al. studied healthy students (aged 20-39 years) and found a 16.4% increase in adiponectin levels, from 8.32±3.32 µg/mL to 9.68±3.83 µg/mL, after six weeks of 60-minute weekly yoga sessions including asanas, pranayama, and meditation [51].

These studies demonstrate that yoga significantly increases adiponectin levels. Supriya et al. demonstrated a substantial 20.1% increase in adiponectin, suggesting the effectiveness of long-term yoga practice in managing MetS and high normal blood pressure [43]. Sarvottam et al. reported a notable 26.5% increase in adiponectin among overweight and obese men over just 10 days [51]. The high improvement rate in this short-term study may be attributed to the intensive daily two-hour yoga sessions combined with psychosocial support through group discussions, lectures, and individual counseling. The studies also indicated enhanced effects when yoga was combined with dietary modifications or whey protein intake [46-48].

Other outcome

One study reported an increase in leptin in the yoga practice group, contrary to the general trend [52]. Telles et al. conducted a randomized comparative trial on overweight and obese individuals (BMI ≥25 kg/m², n=68), and found a 26.8% increase in leptin levels, from 12.3±3.2 ng/mL to 15.6±4.1 ng/mL. The yoga protocol consisted of 45-minute sessions practiced twice daily, combined with a plant-based diet (1650 kcal/day). Additionally, the study found reductions in cholesterol, LDL, lean mass, and body water. These inconsistencies underscore the need for further research in this area.

Discussion

**Figure 2:**
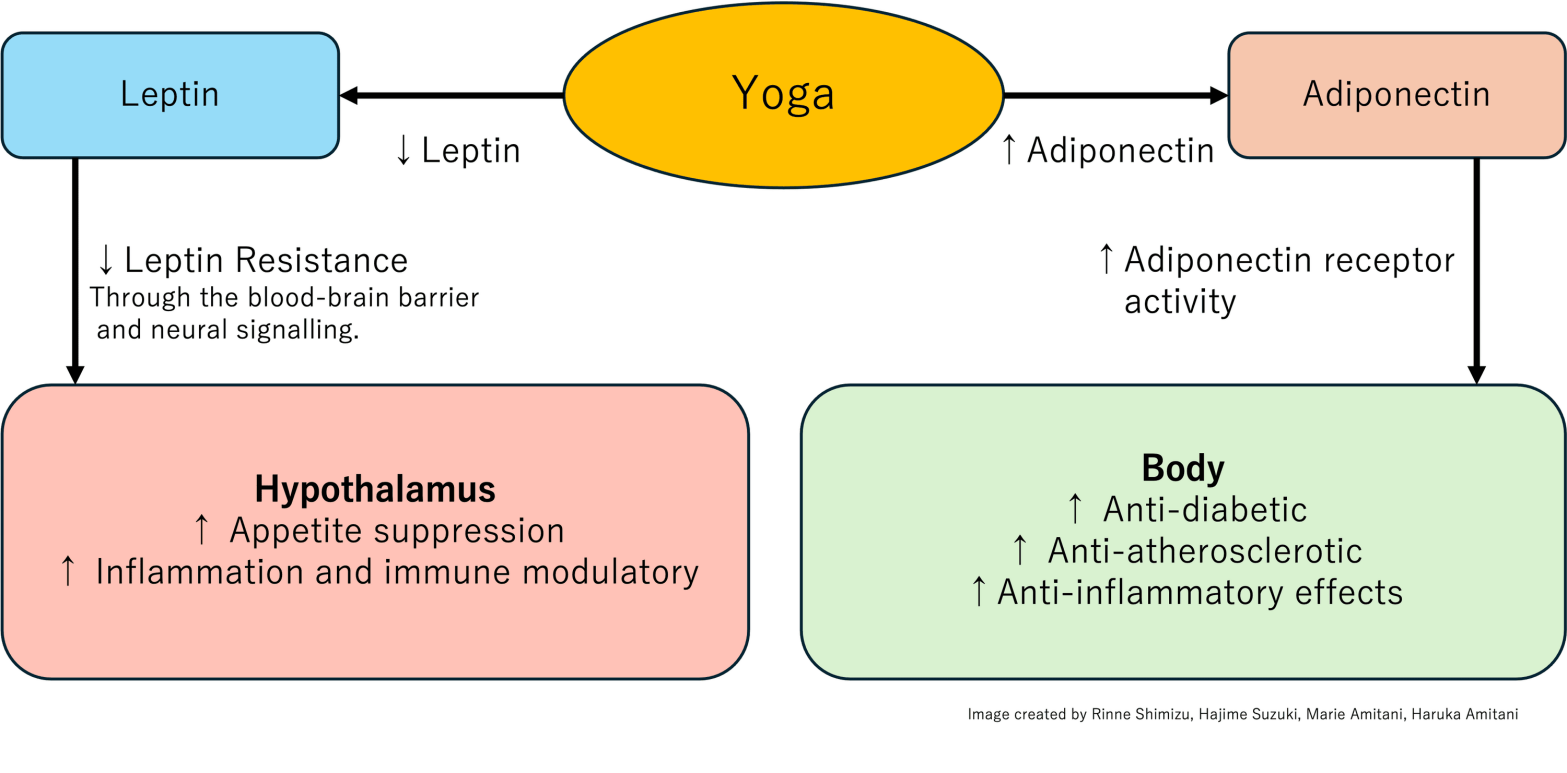
Yoga's Impact on Metabolic Regulation: Leptin and Adiponectin Pathways This diagram illustrates the effects of yoga practice on leptin and adiponectin, two key adipokines involved in metabolic regulation. Yoga is shown to decrease leptin levels while increasing adiponectin levels. Reduced leptin leads to decreased leptin resistance, enhancing its passage through the BBB and neural signaling. This results in increased appetite suppression and modulation of inflammation and immune responses in the hypothalamus. Conversely, increased adiponectin levels enhance adiponectin receptor activity, promoting anti-diabetic, anti-atherosclerotic, and anti-inflammatory effects throughout the body. Arrows indicate the direction of change (↑ increase, ↓ decrease) in response to yoga practice. BBB, blood-brain barrier

Based on the findings from these studies, yoga has been demonstrated to reduce leptin levels while increasing adiponectin levels. A decrease in leptin reduces leptin resistance, facilitating its transport across the BBB and enhancing neural signaling. This, in turn, results in greater appetite suppression and modulation of inflammation and immune responses within the hypothalamus. Conversely, an increase in adiponectin levels enhances adiponectin receptor activity, promoting anti-diabetic, anti-atherosclerotic, and anti-inflammatory effects throughout the body. These findings suggest that yoga may confer potential anti-obesity effects and ameliorate obesity-related chronic inflammation through the regulation of adipokines (Figure 2).

## Conclusions

This review demonstrates that yoga can effectively regulate adipocytokines such as leptin and adiponectin, suggesting potential anti-obesity effects and improvement in obesity-related chronic inflammation. Notably, adipocytokine regulation appeared to be particularly beneficial when yoga was combined with dietary interventions, when practiced intensively for two or more hours daily, or when supplemented with psychosocial support such as group discussions, lectures, and individual counseling. Illustrates the effects of yoga on leptin and adiponectin signaling pathways, depicting how yoga practice can decrease leptin levels while increasing adiponectin levels, leading to improved metabolic regulation. However, there was considerable variation in the duration and style of yoga interventions across studies. Recent years have seen growing interest in the development of anti-obesity drugs targeting leptin and adiponectin. In this context, yoga emerges as a cost-effective and non-invasive intervention that could prove highly beneficial. As research in this field continues to accumulate, we can expect a clearer understanding of yoga's role in regulating adipocytokines and its potential as a complementary approach to managing obesity and related metabolic disorders.
